# Recent Analytical Advances for Decoding Metabolic Reprogramming in Lung Cancer

**DOI:** 10.3390/metabo13101037

**Published:** 2023-09-26

**Authors:** Atiah H. Almalki

**Affiliations:** 1 Department of Pharmaceutical Chemistry, College of Pharmacy, Taif University, P.O. Box 11099, Taif 21944, Saudi Arabia; ahalmalki@tu.edu.sa; 2 Addiction and Neuroscience Research Unit, Health Science Campus, Taif University, P.O. Box 11099, Taif 21944, Saudi Arabia

**Keywords:** metabolomics, lung cancer, NMR, GC-MS, LC-MS, MSI

## Abstract

Lung cancer is the leading cause of cancer-related death worldwide. Metabolic reprogramming is a fundamental trait associated with lung cancer development that fuels tumor proliferation and survival. Monitoring such metabolic pathways and their intermediate metabolites can provide new avenues concerning treatment strategies, and the identification of prognostic biomarkers that could be utilized to monitor drug responses in clinical practice. In this review, recent trends in the analytical techniques used for metabolome mapping of lung cancer are capitalized. These techniques include nuclear magnetic resonance (NMR), gas chromatography–mass spectrometry (GC-MS), liquid chromatography–mass spectrometry (LC-MS), and imaging mass spectrometry (MSI). The advantages and limitations of the application of each technique for monitoring the metabolite class or type are also highlighted. Moreover, their potential applications in the analysis of many biological samples will be evaluated.

## 1. Introduction

Metabolomics is a comprehensive measurement of the repertoire of small molecules, i.e., metabolites, in a biological system [1]. It can be subdivided into two main complementary categories: untargeted metabolomics, which allows a more comprehensive evaluation of as many metabolites as possible in biological systems, and targeted metabolomics, where the research focuses on a set of predefined metabolites for quantitation [2]. Among the different analytical technologies used in metabolomics studies, the utilization of high-throughput instruments, such as nuclear magnetic resonance (NMR) and mass spectrometry (MS), is imperative for their universal detection and excellent identification capacities. Furthermore, the hyphenation of chromatographic methods, such as gas chromatography (GC) or liquid chromatography (LC) with MS detection, offers a significant advantage, allowing the separation of different types of metabolites with reduced matrix effects [3]. However, the performance of GC/LC-MS and NMR for absolute metabolites quantification remain challenging. NMR can quantify metabolites with high reproducibility but relatively low sensitivity, which required concentrations in the micromolar levels. In contrast, MS permits sensitive detection of metabolites up to pico- and nanomolar concentrations suffer from the need for large-scale external calibration or isotopically labeled internal standards, in addition to the confounding matrix effects [4]. Hence, a combination of NMR and MS can provide complementary information to broaden the level of metabolite coverage with adequate sensitivity [5,6].

Lung cancer is the leading cause of cancer-related death worldwide with 1.79 million deaths, amounting to 18% of total deaths due to cancer in 2020 [7]. On a molecular level, metabolic reprogramming is a fundamental trait associated with cancer development to fuel the proliferation and survival of the tumor [8]. The Warburg effect, an increase in Krebs cycle intermediates, and glutamine exploitation are common metabolic traits observed in the lung for its survival [9,10,11]. Monitoring of such metabolic pathways and their intermediate metabolites during treatment courses can provide new avenues concerning the mechanisms of drug actions and treatment strategies, in addition to the identification of prognostic biomarkers that could be utilized in monitoring drug responses in clinical practice [12,13]. Hence, metabolomics has been applied extensively in cancer biology to unveil biomarkers for diagnosis, classifications, and monitoring therapy, as they can provide useful insight into what is currently taking place inside the cells [14]. In lung cancer, metabolomics experiments have unraveled different biomarkers that discriminate lung cancer patients from healthy controls [15], predict lung cancer risk in smokers [16] and discriminate lung cancer from chronic respiratory disease [17]. Moreover, metabolomics studies have been employed to determine the rewired metabolic pathways in lung cancer disease viz. up-regulation and reprogramming of glycolysis and TCA [9], up-regulation of phospholipid metabolic pathways and fatty synthesis [18], and suppression of the oxidative pentose pathway [19]. However, few metabolomics studies have been applied to monitor lung cancer therapy and discover novel prognostic biomarkers during the treatment course [20].

In this review, we do not aim to completely cover every single aspect of metabolomics analysis, such as sample pretreatment methods and data analysis strategies, as these items have been recently covered [21,22,23,24,25,26]. Instead, the recent trends in measuring the lung cancer metabolome will be highlighted concerning their metabolic pathways and potential applications. Moreover, the advantages and limitations concerning the application of each technique towards monitoring which metabolite class or type will also be emphasized.

## 2. NMR Metabolomics in Lung Cancer Research

**Figure 1:**
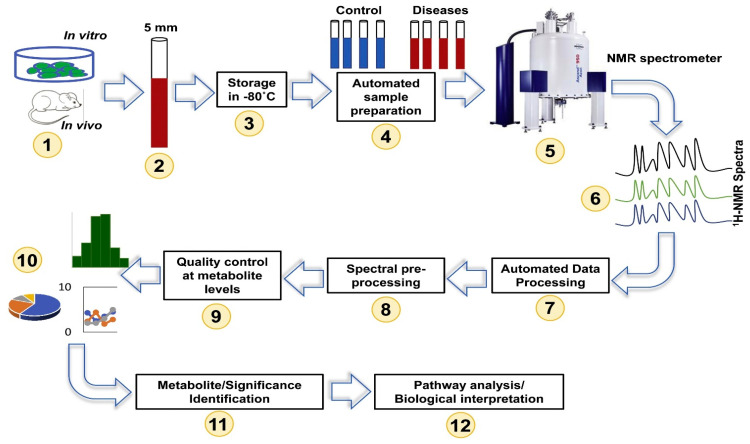
Overview of NMR-based metabolomics workflow. Reprinted with permission from Ref. [40].

NMR spectroscopy provides rapid, unbiased metabolite detection capabilities in addition to high-throughput and quantitative properties. Although NMR is a less sensitive technique, and requires more expensive instrumentation compared to MS, its major advantages lie in its high repeatability, noninvasiveness, and minimum sample preparation procedures [27]. Such advantages allow visualization of the actual metabolic state of the studied living organism at a particular point in time [28]. Hence, NMR metabolomics has been reported extensively in analyzing many biological specimens, including serum [29], plasma [30], tissues [31], cerebrospinal fluid [32], amniotic fluid [33], seminal fluid [34], and fecal extracts [35], as well as cell lysates and cell growth media [36]. The most common pulse sequences used in ^1^D NMR-based metabolomics studies include the ^1^D NOESY, ^1^H CPMG (Carr–Purcell–Meiboom–Gill), and ^1^H diffusion-edited [28,37]. Employing these sequences allows the detection of low and high molecular weight metabolites, which are particularly useful in biofluids such as serum/plasma. However, the analysis of NMR spectra can be challenging due to signal overlap, which limits the number of compounds that can be unambiguously identified and quantified [38]. To overcome these challenges, advanced NMR techniques like two-dimensional (2D) NMR spectroscopy have been developed to enable the separation of the congested signals in a secondary frequency domain by transferring magnetization between nuclei of the same type, as seen in correlated spectroscopy (COSY) and total correlated spectroscopy (TOCSY), or between different types of nuclei, such as multiple bond correlation experiments using heteronuclear nuclei (HMBC) and single quantum coherence spectroscopy (HSQC) [39]. Such acquisitions of more detailed and resolved spectra enable the identification and quantification of a larger number of metabolites. A detailed workflow of NMR-based metabolomics experiments is presented in Figure 1.

Hence, NMR-based metabolomics has become a powerful clinical tool in precision oncology, particularly lung cancer research (Table 1) [40]. For example, a study by Fan et al. utilized stable isotope-resolved metabolomics (SIRM) to investigate metabolic changes in human lung cancer patients [9]. They infused uniformly labeled ^13^C-glucose into patients and analyzed the metabolic profiles of lung cancer tissues and paired non-cancerous lung tissues using NMR and GC-MS. This study highlighted the altered regulation of metabolic pathways in lung cancer, such as anaplerotic pyruvate carboxylation, more active glycolysis, and Krebs cycle via tracing the carbon flux through these metabolic pathways [9]. In addition, NMR-based metabolomics has shown promise in the identification of diagnostic biomarkers. For example, a study by Chung et al. used ^1^H-NMR-based serum metabolomics in combination with ^18^F-FDG PET/CT to diagnose metastatic lung cancers [41]. They identified potentially increased pyruvate carboxylase and high proliferation rates of metastatic tumors that could be associated with high utilization of pyruvate, leading to a decrease in the serum pyruvate level. This is in line with another study by Sarlinova et al., which demonstrated significant increases in glucose, citrate, acetate, 3-hydroxybutyrate, and creatinine, and decreases in pyruvate, lactate, alanine, tyrosine, and tryptophan in cancer patients compared with healthy control subjects, posing the potential of NMR plasma metabolomics as a screening tool for lung cancer and showing promising statistical discrimination against healthy controls [42]. The use of ^1^H-NMR spectroscopy aids in the identification of serum biomarkers for detecting NSLC at an early stage, distinguishing it from healthy individuals. A group of 18 metabolites, including organic acids, amino acids, alcohols, lipids, and molecules involved in lipid metabolism, were observed to have significant differences between the two groups [43]. ^1^H-NMR-based metabolomics shows promising capabilities in distinguishing lung cancer from those with chronic obstructive pulmonary disease (COPD), even in the early stages of lung cancer [17].

NMR-based metabolomics approaches show excellent potential in identifying prognostic metabolites during the course of treatment. For example, ^1^H-NMR identified the changes in pre-and postoperative plasma metabolites of 74 patients diagnosed with resectable stage I-IIIA NSCLC, revealing a significant increase in lactate, cysteine, and asparagine levels and decreased acetate levels in the postoperative plasma patients [44]. In addition, ^1^H-NMR-based metabolomics has been employed to assess the response to immune checkpoint inhibitors in patients with NSCLC, revealing high levels of critical metabolites such as pyruvate and alanine in non-responder subjects [37]. Another study by Hao et al. analyzes ^1^H-NMR and GC-MS serum metabolomic profiles of 25 lung cancer patients undergoing chemotherapy and/or radiation to evaluate the feasibility of metabolites as temporal biomarkers of clinical outcomes [45].

High-resolution magic angle spinning (HR-MAS) NMR spectroscopy has emerged as a powerful tool for investigating tissue metabolism, including in the context of lung cancer metabolomics [46]. This technique has been used to study the metabolomic characteristics of lung tissues from patients with lung cancer, revealing important metabolic alterations associated with the disease such as high levels of aspartate, phosphocholine, glycerophosphocholine, and lactate, and significantly low levels of glucose and valine in cancer tissues compared to non-cancerous counterparts [46]. Another investigation utilized HR-MAS NMR spectroscopy to compare the metabolite composition between NSCLC cell lines that were induced with cisplatin resistance and their subsequently de-induced counterparts [47]. The metabolites primarily altered in both the cisplatin-resistant cells and their de-induced counterparts include glutathione and taurine, suggesting these metabolites could serve as significant biomarkers for identifying cisplatin resistance. These identified metabolites, such as those involved in glutathione synthesis, indicate potential mechanisms of resistance that may be targeted by modified drugs or novel combinations to combat this form of resistance [47]. Additionally, HR-MAS NMR spectroscopy was utilized to investigate the metabolic changes in A549 human lung cells in response to cisplatin exposure, both in vitro and ex vivo. The results revealed an increase in unsaturated triglycerides and nucleotide sugars in the cisplatin-treated cells, indicating their potential as biomarkers for treatment response. [20].

**Table 1:** Summary of the NMR metabolomics studies investigating lung cancer.

No.	Aim of the Study	Sample	Participants	Altered Metabolites Associated with Lung Cancer	Ref.
1	Investigating the altered metabolic pathways in lung cancer	Tissues	12	↑ Alanine ↑ Lactate ↑ Glutamic acid	[9]
2	Investigating the metabolomic changes in primary and secondary lung cancer patients vs. control	Plasma	256	↑ Glucose, ↑ Citrate, ↑ Acetate, ↑ Hydroxybutyrate, and↑ Creatinine, ↓ Pyruvate, ↓ Tyrosine, ↓ Tryptophan	[42]
3	Biomarker discovery to support the early diagnosis and prognosis of NSCLC	Serum	269	↑ Leucine, ↑ Acetate, ↑ Glutamate, ↑ Creatine, ↑ Lactate, ↓ Adipic acid,	[43]
4	Investigating the metabolomic changes after complete NSCLC removal	Plasma	74	↑ Lactate, ↑ Cysteine, ↑ Asparagine, ↓ Acetate	[44]
5	Investigating the response to immune checkpoint inhibitors in patients with NSCLC	Serum	50	↑Pyruvate, ↑ Alanine	[37]
6	Investigating the metabolic disturbances and metabolites of diagnostic potential in lung cancer	Serum	81	↓ Histidine, ↓ Glutamine, ↓ Glycine, ↓ Threonine, ↓ Alanine, ↓ Valine	[48]
7	Investigating lung cancer metabolic signatures in urine and assessing the diagnostic potential of this approach	Urine	125	↑ N-Acetylglutamine, ↑ Hydroxyisobutyrate, ↑ Creatinine, ↓ Trigonelline, ↓ Hippurate	[49]
8	Investigating the variations in the metabolicprofile of lung cancer patients and healthy control	Plasma	163	↑ Pyruvate, ↑ Lactate, ↓ Glucose, ↓ Citrate, ↓ Acetate, ↓ Formate, ↓ Methanol, ↓ Histidine, ↓ Glutamine, ↓ Tyrosine, ↓ Alanine	[50]
9	Investigating metabolomic characteristics and identifying possible biomarkers in lung tissue	Tissues	17	↑ aspartate, ↑ phosphocholine, ↑ glycerophosphocholine, ↑ lactate, ↓glucose, ↓ valine	[46]
10	Investigate the metabolic changes in A549 human lung cells in response to cisplatin exposure	A549 Cell line	--	↓ unsaturated triglycerides, ↓ nucleotide sugars	[20]

## 3. GC-MS Metabolomics in Lung Cancer Research

The use of gas chromatography–mass spectrometry (GC-MS) in the realm of lung cancer metabolomics has emerged as a crucial methodology for identifying biomarkers and altered metabolic pathways in patients with lung cancer (Table 2). GC-MS has proven to be highly effective at providing both quantitative and qualitative analysis, particularly for volatile organic compounds (VOCs) with high sensitivity and specificity, posing these compounds as reliable indicators for diagnosing and predicting lung cancer [51,52]. Hence, several studies have used GC-MS to analyze VOCs present in the exhaled breath of patients with lung cancer and healthy controls [53,54]. For example, GC-MS was applied to analyze the breath exhaled by 107 patients with lung cancer and 29 healthy controls, and identified concentrations of 56 VOCs [54]. Four target VOCs were selected for further analysis, including nonanal, acetoin, acetic acid, and propanoic acid. The concentrations of these VOCs were significantly different between patients with lung cancer and healthy controls. In addition, GC-MS analysis from 65 individuals with lung cancer to those of 31 healthy participants showed variations in the levels of over 50 substances. Utilizing a combination of these different VOCs, achievable sensitivity rates ranging from 52% to 80% were obtained, while maintaining a specificity rate of 100% for detecting lung cancer patients [53]. Interestingly, GC-MS has also been employed in detecting VOCs released from lung cancer cells (A549) and non-cancerous lung cells (WI38VA13), identifying decane and heneicosane as noticeable VOCs produced from the cancerous cell line (A549), whereas non-cancerous WI38VA13 cells emitted 1-Heptanol and heptadecane, suggesting the potential employment of these metabolites as a non-invasive screening methodology for lung cancer [55].

Comprehensive two-dimensional gas chromatography coupled with mass spectrometry (GC × GC-MS) has played a significant role in the analysis of complex mixtures of VOCs [56]. This technique uses a dual-column system, with one column being nonpolar to separate analytes based on their volatilities and the other column being polar to increase peak capacities. The columns are interconnected through a modulator that selectively traps and releases analytes from the primary column onto the secondary column, thus enhancing separation capabilities using a 2D approach [57]. Hence, two-dimensional gas chromatography has proven to be a valuable technique for the identification and quantification of VOCs in patients with lung cancer [58,59]. For example, this approach was employed to reveal the metabolic abnormalities in the volatile content of human breath of lung cancer patients and healthy volunteers. Interestingly, overexpression of fatty acid methyl ester and ketone compounds was observed in cancer patients which could be attributed to the inflammation processes inside the lungs [58].

The identification and quantification of VOCs using GC-MS or GC × GC-MS can be challenging because of the low concentrations of these compounds in patients’ samples [60]. However, advancements in extraction and preconcentration techniques, such as needle trap and solid phase microextraction (SPME) devices, have enabled the detection and analysis of low-level VOCs in breath samples from patients with lung cancer [61,62]. Furthermore, the on-fiber-derivatization SPME-GC/MS method has been used to assess straight aldehydes C3–C9 in exhaled breath samples from patients with NSCLC. The SPME fiber was loaded with pentafluorobenzyl hydroxylamine as a derivatization reagent for the aldehydes, and various extraction times were examined. This technique demonstrated favorable accuracy, precision, and sensitivity in detecting aldehydes in exhaled breath specimens. In addition, advancements in nanoparticle technology have contributed to the progress in this area. Specifically, the use of single-walled carbon nanotubes coated with non-polymeric organic materials as preconcentration kits has enabled the detection of lung cancer biomarkers in breath samples through GC-MS analysis [63].

On the other hand, GC-MS has also been applied to analyze primary metabolites in the serum of patients with lung cancer, providing valuable insights into the metabolic changes associated with this disease [45]. The identified metabolites, including hydroxylamine, tridecan-1-ol, and octadecan-1-ol, were associated with survival in lung cancer patients. In contrast, other primary metabolites such as tagatose, hydroxylamine, glucopyranose, and threonine have shown potential as biomarkers for disease progression [45]. Another study examined the plasma metabolome of individuals with lung cancer by GC-MS, identifying notable changes in the levels of organic acids such as pipecolic acid, 3-hydroxybutyric acid, and uric acid, in addition to amino acids such as alanine, glycine, glutamine, threonine, and 5-hydroxytryptophan, and fatty acids such as palmitic acid [64]. Furthermore, GC-MS metabolomic analysis of primary metabolites in blood samples has also been employed for the detection of lung cancer. This approach successfully identified specific metabolites that demonstrated significant differences between patients with lung cancer and controls. For instance, elevated levels of maltose, glycerol, palmitic acid, glutamic acid, lactic acid, and ethanolamine were observed in lung cancer samples compared with controls. Conversely, decreased levels of amino acids such as lysine, tryptophan, and histidine were found in patients with lung cancer. These findings highlight the potential value of metabolomics in diagnosing lung cancer and monitoring treatment progress [65]. Nevertheless, a significant limitation in the analysis of these metabolites using GC-MS is the requirement for robust derivatization techniques that enhance the volatility and stability of these compounds [66]. Such derivatization techniques include silylation and methylation to convert non-volatile or polar compounds into more volatile and stable derivatives, making them suitable for GC-MS analysis [67]. Such pre-sample treatment steps could aid with discrepancies in the analysis results, such as glutamate, which was found elevated in the blood of lung cancer patients by Fahrmann et al. [68], whereas Hori et al. [69] reported a decreased level of glutamate, posing the need for a robust metabolomics approach when analyzing this class of metabolites.

**Table 2:** Summary of the GC-MS metabolomics studies investigating lung cancer.

No.	Aim of the Study	Sample	Participants	Altered Metabolites Associated with Lung Cancer	Ref.
1	Developing a non-invasive lung diagnostic method for detection of lung cancer	Breath	96	↓ Isoprene, ↓ Acetone, ↓ Methanol	[53]
2	Investigating the difference of VOCs in breath exhaled by patients with lung cancer from healthy control and after resection surgery	Breath	136	↑ Nonanal, ↑ Acetoin, ↑ Acetic acid, ↑ Propanoic acid	[54]
3	Identifying VOC biomarkers in patients with NSCLC vs. healthy smokers, non-smokers, and patients with COPD	Breath	136	↑ 2-Methylpentane, ↑ Isoprene, ↓ Ethylbenzene, ↓ Styrene	[70]
4	Investigating the potential of GC × GC-MS for lung cancer screening	Breath	29	↑ Fatty acid methyl esters, ↑ Ketones	[58]
5	Investigating the use of needle trap device with GC-MS for assessment of asthma, COPD and lung cancer patients VOCs	Breath	56	↑ 2-Propanol, ↑ Undecane, ↑ 4-Methyl Octane, ↑ Dodecanone, ↑ 3-Amino butanoic Acid, ↑ Nonanal	[61]
6	Investigating the difference of VOCs in breath exhaled by patients with lung cancer from healthy control	Breath	53	↑ Propane, ↑ Carbon disulfide, ↓ 2-Propenal, ↓ Ethylbenzene, ↑ Isopropyl alcohol	[62]
7	Studying VOCs emitted by the in vitro cultured human lung cancer cells and non-cancerous lung cells	(A549), (WI38VA13) Cell lines	--	↑ Decane, ↑ Heneicosane, ↓ 1-Heptanol, ↓ Heptadecane	[55]
8	Evaluating prognostic markers of clinical outcomes for lung cancer patients undergoing chemotherapy and/or radiation treatment	Serum	25	↓ Hydroxylamine, ↓ Tridecan-1-ol, ↓ Octadecan-1-ol, ↑ Tagatose, ↑ Hydroxylamine, ↑ Glucopyranose, ↑ Threonine	[45]
9	Investigating the pathophysiological changes during early lung adenocarcinoma development	Plasma	59	↓ Alanine, ↓ Glutamine, ↓ Glycine, ↓ 5-Hydroxytryptophan, ↓ 3-Hydroxy butyric acid, ↓ Pipecolic acid, ↓ Uric acid, ↑ Palmitic acid	[64]
10	Investigating the difference of primary metabolites in blood of patients with lung cancer from healthy control	Plasma	62	↑ Maltose, ↑ Glycerol, ↑ Palmitic acid, ↑ Glutamic acid, ↑ Lactic acid, ↑ Ethanolamine, ↓ Lysine, ↓ Tryptophan, ↓ Histidine	[65]

## 4. LC-MS Metabolomics in Lung Cancer Research

The combining of LC and MS has become a cornerstone in the field of metabolomics research due to its ability to provide high resolution and sensitivity [71]. Such combinations offer significant advantages viz. low ion suppression effects and highly effective chromatographic separation. Recently, the adoption of ultra-high-performance liquid chromatography techniques (UHPLC) has further enhanced column efficiency, peak resolution, and sensitivity compared to traditional LC-MS methods [72]. Unlike NMR spectroscopy and GC-MS, which have limitations in terms of metabolite coverage, LC-MS allows for broader analysis thanks to the availability of diverse chromatographic columns with varied characteristics like different polarities and different MS ionization modes. RP columns such as C8 and C18 are often utilized when measuring weakly polar or non-polar metabolites, whereas HILIC columns are preferred for analyzing polar metabolites [73]. Currently, LC-MS is widely embraced within the cancer metabolomics field due to its desirable attributes including excellent reproducibility and stability during various metabolic studies.

**Figure 2:**
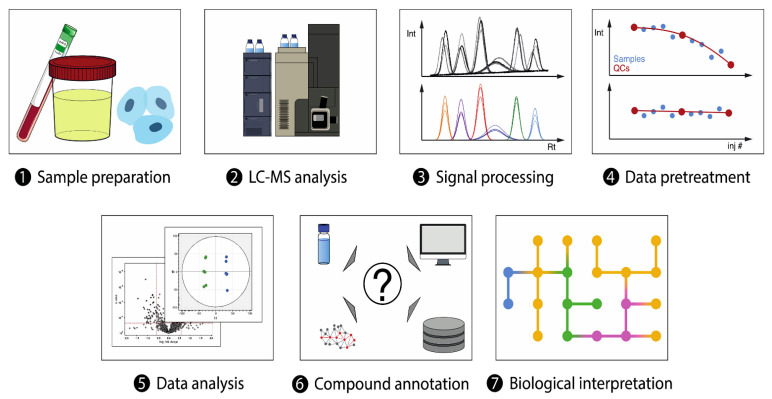
Overview of LC−MS-based untargeted metabolomics workflow. Reprinted with permission from Ref. [74].

There are currently two primary approaches in lung cancer metabolomics research that utilize LC-MS: untargeted and targeted. The untargeted approach involves the comprehensive, unsupervised analysis of all metabolites in a sample (Figure 2) [74]. It is inevitable to use high-resolution MS detectors like time-of-flight or orbitraps for this type of analysis to ensure the identity of the detected metabolites [75]. This approach has been applied extensively in lung cancer research to identify potential biomarkers for early detection, prognosis, and treatment response, as well as the altered metabolic pathways (Table 3). For example, LC-MS orbitrap-based global metabolomic approach has been adopted to examine the serum of early-stage NSCLC patients [11]. The analysis revealed several significant metabolites belonging to various classes such as acylcarnitines, organic acids, and amino acids. Remarkably, these metabolites demonstrated a strong discrimination ability when evaluated using a multivariate ROC curve with an AUC value of 0.836 between the patient group and their corresponding control group [11]. The same technology has been also utilized to characterize KRAS mutants in NSCLC cells [76], and also to compare two metabolomes (plasma and serum) of small-cell lung cancer (SCLC) patients undergoing treatment with standard chemotherapy [77], as well as to detect the metabolic alteration in the serum of NSCLS patients identifying several perturbed pathways such phenylalanine metabolism, linoleic acid metabolism, and biosynthesis of bile acids [78]. Quadrupole time of flight mass spectrometry (QTOF-MS) analyzer is another advanced and cost-effective technology that has been combined with LC in numerous untargeted metabolomics studies, particularly in the field of lung cancer research. For example, LC-QTOF-MS was utilized to analyze the metabolic profiles of different histological subtypes of NSCLS, using both RP and HILIC columns [79]. The analysis of surgically resected lung cancer tissues from NSCLC patients using this developed methodology revealed increased levels of acylcarnitines, fatty acids, phospholipids, and amino acids compared to control tissues [79]. UHPLC-QTOF-MS also has been applied for untargeted analysis of lung cancer serum samples compared to healthy control groups identifying differential metabolites related to lipid metabolism such as choline, free fatty acids, and lysophosphatidylcholines [80].

Lung cancer research has also utilized targeted metabolomics approaches that involve the measurement and quantification of specific metabolites or classes of metabolites related to the altered metabolic pathways (Table 3). To this end, mass spectrometry-based approaches, particularly triple quadrupole mass spectrometry coupled to LC (LC-QqQ-MS) with multiple reaction monitoring (MRM) transitions have been commonly used for a focused analysis of these metabolites. LC-QqQ-MS was employed to investigate the changes in free amino acid profiles in patients with NSCLS as a targeted approach. These studies identified abnormalities in the blood profiles of certain free amino acids that have potential diagnostic value for NSCLC [81,82,83]. It is important to mention that when analyzing amino acids using LC-MS, a precolumn derivatization step is typically necessary for increased sensitivity [84]. One method to accomplish this is by using the iTRAQ^®^-LC-MS/MS with 42 internal standards of physiological amino acids and amines as an isobaric tagging reagent, allowing for absolute quantification of these amino acids through isotope ratio analysis [85]. Organic acids are another class of metabolites that have been targeted in lung cancer research using LC-MS. For example, a study utilized LC-QqQ-MS to analyze six low-molecular-weight organic acids, including fumaric glutaric acid, pyroglutamic acid, citric acid, lactic acid, and succinic acid in lung cancer patients [86]. Among the studied acids, pyroglutamic showed the most promising discriminatory potential and accurately distinguished between NSCLC patients and control subjects. LC-QqQ-MS analysis was also used to identify elevated differential metabolites levels related to fatty acid metabolism, such as arachidonic acid, linoleic acid, and their intermediate hydroxyeicosatetraenoic acids, in lung cancer serum samples compared to healthy control groups [87].

**Table 3:** Summary of the LC-MS metabolomics studies investigating lung cancer.

No.	Aim of the Study	Sample	Participants	Altered Metabolites Associated with Lung Cancer	Ref.
1	Applying LC-MS orbitrap-based global metabolomic approach for studying NSCLC potential markers	Serum	75	↓ Histidine, ↑ Carnitine, ↓ Malic acid, ↓ Methionine, ↓ pyroglutamic acid, ↓ Leucine, ↓ Tyrosine	[11]
2	Comparing plasma and serum metabolomes of SCLC patients undergoing treatment with standard chemotherapy	Serum Plasma	29	No significant difference between the two biofluids	[77]
3	Characterize the metabolic alteration of NSCLC and biomarkers discovery	Serum	436	↑ Hypoxanthine, ↑Glycoursodeoxycholic acid, ↓ Linoleic acid, ↓ 2,4-Dihydroxybenzoic acid, ↓ Testosterone sulfate, ↓ Choline, ↓ Piperine	[78]
4	Development of an LC-QTOF-MS method that could discriminate NSCLC histological subtypes	Tissue	15	↑ Acylcarnitines, ↑ Fatty acids, ↑ Phospholipids, ↑ Amino acids	[79]
5	Identifying metabolites differentially regulated in lung cancer from healthy controls	Serum	46	↓ Choline, ↓ Linoleic Acid, ↑ Lysophosphatidylcholines	[80]
6	Investigating plasma free amino acids for detecting lung cancer	Plasma	4020	↑ Proline, ↑ Isoleucine, ↑ Ornithine, ↓ Glutamine, ↓ Histidine, ↓ Tryptophan	[81]
7	Investigating serum organic acids for detecting lung cancer	Serum	152	↑ 2-Hydroxybutyric acid, ↓ Fumaric acid, ↓ Lactic acid, ↓ Pyroglutamic acid	[86]
8	Investigating the role of free fatty acids in lung cancer development	Serum	220	↑ Arachidonic acid, ↑ linoleic acid, ↑ Hydroxyeicosatetraenoic acids	[87]
9	Identifying potential plasma biomarkers for NSCL	Plasma	211	↑ Cortisol, ↑ Cortisone, ↓ 4-Methoxyphenylacetic acid	[88]
10	Identifying potential biomarker for lung cancer early detection	Urine	1005	↑ Creatine riboside, ↑N-Acetylneuraminic acid	[89]

## 5. Mass Spectrometry Imaging in Lung Cancer Research

MS imaging (MSI) is a valuable technology for the spatial characterization of biological molecules in tissue sections through direct ionization and detection [90]. This technique often incorporates matrix-assisted laser desorption/ionization (MALDI), which gently ionizes various biological molecules. A typical MALDI-MSI analysis achieves spatial resolutions ranging from 10 to 200 μm, with resolution primarily determined by the size of the laser-treated region, typically exceeding 5 µm. However, the application of MALDI-MSI can be challenging due to many factors such as diffusion of metabolites during matrix application and variations in crystal formation, which may limit the spatial resolution. Additionally, analysis of low-molecular-weight metabolites using MALDI-MS has been limited because various matrix compounds and their ion peaks interfere with detection. As a result, lipid molecules have emerged as preferable targets for MSI studies since they have higher m/z ranges compared to other metabolites [91]. Furthermore, lipids are abundant in tissues and readily ionized due to their polar head group composition. Hence, MALDI-MSI lipidomics approaches have been utilized to investigate and characterize the lipid profiles of lung cancer tissues. For example, matrix-assisted laser desorption/ionization imaging mass spectrometry (MALDI-MSI) has been used to identify and localize specific phospholipids within lung cancer tissue, revealing significant differences between tumor and adjacent healthy tissue with a substantial increase in the selected phospholipids species in cancer regions [92]. Similarly, numerous studies used MALDI-MSI-based metabolomics to identify variances between NSCLC tumors and normal lung regions through lipidomic analysis [93,94,95]. MALDI-MSI was also utilized to validate lipidomic variations among different subtypes of NSCLC [96,97]. Additionally, MALDI-MSI was utilized to elucidate therapeutic effects of neoadjuvant chemotherapy in patients with NSLC, identifying alterations in lipid metabolism as a potential indicator of treatment response [98]. Among all the analyzed lipids, sphingomyelin-specifically SM d18:1/15:0 or d16:1/17:0-was exemplified as a prognostic marker in individuals with NSCLC. It was found that high mass intensity for SM correlated significantly with favorable prognosis [98]. These findings demonstrate the utility of MALDI-MSI in investigating the changed metabolic processes observed in lung cancer, particularly through the examination of the differential lipids.

## 6. Conclusions and Future Perspectives

Lung cancer-based metabolomics studies have utilized various analytical techniques, including NMR, GC-MS, LC-MS, and MSI. Each of these techniques offers unique advantages and limitations in terms of sensitivity, selectivity, and spatial resolution. NMR is a powerful technique for non-targeted metabolite profiling and allows for the identification and quantification of many metabolites with excellent reproducibility and high-throughput analysis. However, its limited sensitivity and inability to detect low-abundance metabolites restrict its application in certain metabolomics studies. In addition, signal overlapping and spectral complexity can pose challenges in identifying significant metabolites. Hence, emphasis should be placed on multidimensional techniques such 2D and even 3D NMR experiments in metabolomics lung cancer research. Additionally, employing complementary techniques such as GC-MS seems inevitable, particularly for analyzing another class of metabolites, i.e., VOCs. The peak capacities and resolution of GC-MS analysis can be also enhanced using two-dimensional chromatography setups such as GC × GC-MS providing higher separation capabilities and improved metabolite coverage posing them as powerful techniques in next-generation metabolomics lung cancer research. LC-MS, on the other hand, offers excellent sensitivity and the ability to analyze a wide range of metabolites, thus it has been employed extensively in many untargeted and targeted metabolomics studies. Furthermore, recent advancements in LC-MS technologies, such as the use of high-resolution mass spectrometers, have significantly improved the resolution and coverage of metabolite analysis in lung cancer metabolomics. Another area of emerging technology is the spatial metabolomics approach, which involves the use of MALDI-MSI to visualize the spatial distribution of metabolites in lung cancer tissues. However, such an approach is restricted to analyzing relatively high molecular metabolites, such as lipids, due to a matrix effect. Hence, other ionization techniques, such as secondary ion mass spectrometry and desorption electrospray ionization, should be explored to expand the scope of metabolite analysis in lung cancer tissues. Overall, in metabolomics research, it is important to carefully select the appropriate analytical technique based on the specific goals and requirements of the study.
